# Multiple intracerebral hemorrhages secondary to eosinophilic granulomatosis with polyangiitis: A case report and literature review

**DOI:** 10.1002/kjm2.12882

**Published:** 2024-08-19

**Authors:** Guo Li, Xing‐Ren Liu, Ling‐Jing Yang

**Affiliations:** ^1^ Department of Respiratory and Critical Care Medicine Xichong County People's Hospital Nanchong Sichuan China; ^2^ Department of Respiratory and Critical Care Medicine Sichuan Provincial People's Hospital Chengdu Sichuan China

Eosinophilic granulomatosis with polyangiitis (EGPA) is a type of systemic vasculitis that affects multiple systems. The most common neurological complication is peripheral nervous system disease, while central nervous system involvement is less common, and intracerebral hemorrhage is rare. Few cases of multiple intracerebral hemorrhages have been reported in EGPA patients.

A 55‐year‐old woman presented with dyspnoea, edema in both lower legs, and a 1‐month history of numbness of the left plantar foot. She had been admitted to a local hospital more than 20 days prior for a brief loss of consciousness with urinary incontinence. Her head CT scan revealed no abnormalities. Coronary angiography was performed because of elevated high‐sensitivity troponin levels and did not reveal any vascular issues. The patient had a medical history of sinusitis, nasal polyps, and bronchial asthma spanning over 10 years. She is currently taking intermittent inhaled budesonide/formoterol for asthma control, with attacks occurring every 2–3 years.

The patient presented with an elevated peripheral blood eosinophil count of 6.43 × 10^9^/L (<0.5 × 10^9^/L), a troponin T level of 161 ng/L (<9 ng/L), a D‐dimer level of 4 mg/LFEU (<0.5 mg/LFEU), a BNP level of 380 pg/mL (<100 pg/mL), and an IgE level of 1560 IU/mL (<100 IU/mL). The chest CT findings included scattered small opacities, ground‐glass opacities in both lungs, interlobular septal thickening, and bilateral pleural and pericardial effusion (Figure 1A,B). Further testing of the right pleural effusion confirmed a fluid leak. Ultrasound examination of the lower limb veins revealed thrombosis in the left posterior tibial vein, fibular vein, and bilateral intermuscular calf veins. The patient was diagnosed with suspected pulmonary embolism but was unable to undergo CT pulmonary angiography due to severe wheezing. Treatment with low‐molecular‐weight heparin anticoagulation was initiated.

**FIGURE 1:**
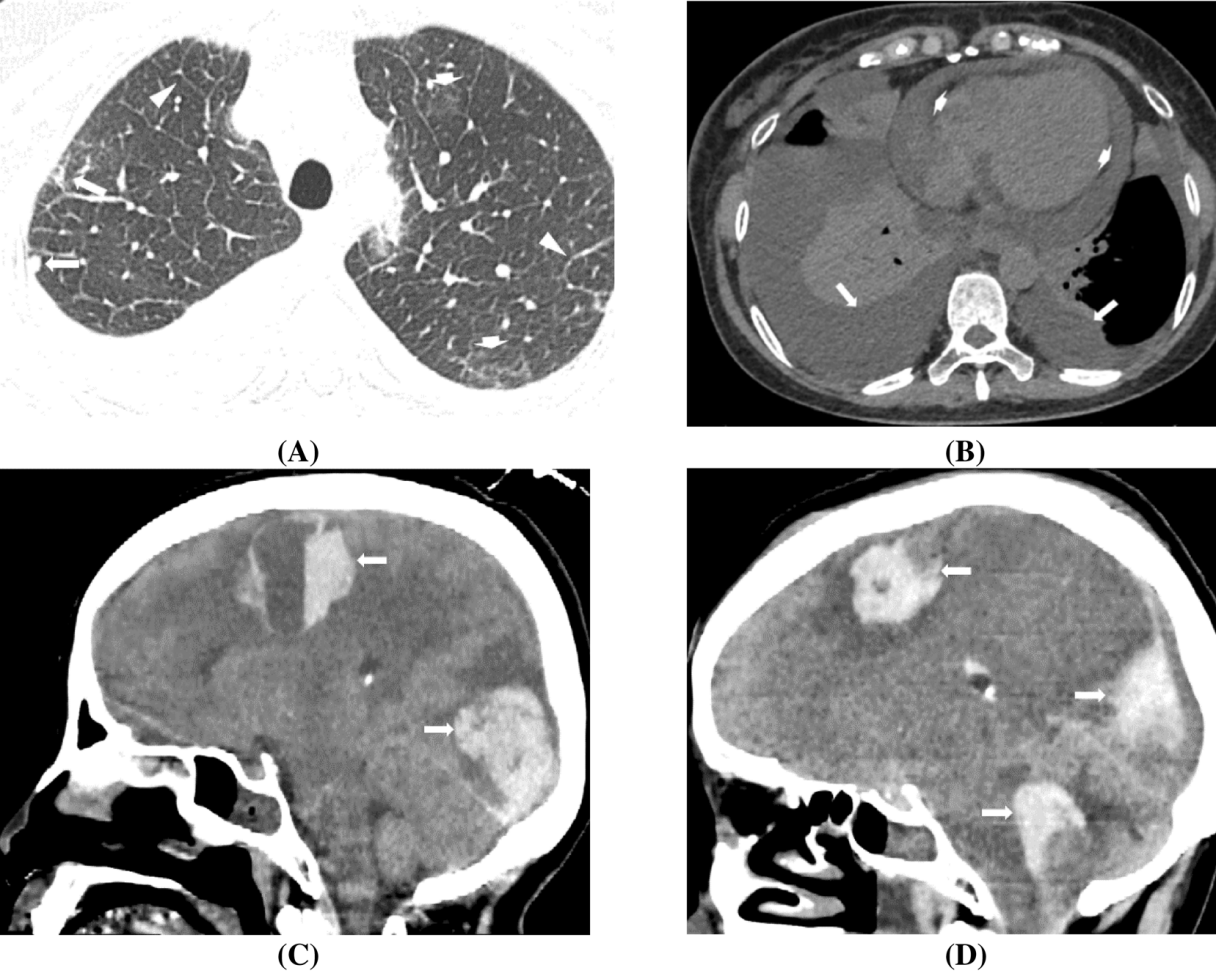
(A) Chest CT lung window revealed scattered small opacities (long arrows) and ground‐glass opacities (short arrows) in both upper lungs, accompanied by interlobular septal thickening (arrowhead). (B) Chest CT mediastinal window showed bilateral pleural effusion (long arrows) and pericardial effusion (short arrows). (C) Head CT revealed two large hematomas in the right frontal and occipital lobes (arrows). (D) Postoperative head CT showed a slight reduction in both previous hematomas but also the formation of a new hematoma (arrows).

Further investigation with routine urinalysis revealed occult blood 1+, protein 2+, and serum p‐ANCA (+), while the results were negative for MPO and PR3 antibodies. Together with the results of electromyography demonstrating neurogenic damage, the patient was diagnosed with EGPA. Following the intravenous infusion of 40 mg of methylprednisolone, the peripheral blood eosinophil count decreased to 0.24 × 10^9^/L, and the D‐dimer level decreased to 1.89 mg/LFEU. However, the patient developed vomiting, unsteadiness, decreased strength in the left limb and a positive Babinski sign on the fifth day after admission. An emergency head CT confirmed multiple hemorrhages in the right frontal and occipital regions (Figure 1C). Despite the active surgical evacuation of the intracranial hematoma, the patient died of recurrent hemorrhage and cerebral herniation (Figure 1D).

The diagnosis of EGPA is primarily determined based on the American College of Rheumatology/European Alliance of Associations for Rheumatology criteria.^1^ Our patient fulfilled four criteria: an eosinophil count ≥1 × 10^9^/L (+5) and positive diagnoses of obstructive airway disease (+3), nasal polyps (+3), and multiple mononeuritis (+1), resulting in a cumulative score of 12 points, which confirmed the EGPA diagnosis.

The involvement of the central nervous system in EGPA can present in four main ways: cerebral ischemic lesions, intracerebral hemorrhages, cranial nerve palsies, and vision loss. Among these, cerebral ischemic lesions are the most common. While intracerebral hemorrhage is less common, it is a significant predictor of poor prognosis.^2^ It is hypothesized that a combination of eosinophil‐induced tissue damage and vasculitis may play a crucial role in this process.

In the literature, a total of two EGPA patients with multiple intracerebral hemorrhages have been reported to have successfully recovered after hormonal and immunosuppressive therapy.^3^, ^4^ In our patient's case, multiple intracerebral hemorrhages occurred after the peripheral blood eosinophil count had normalized, and there were recurrent hemorrhages after surgery. The administration of anticoagulants such as low‐molecular‐weight heparin has been associated with intracerebral hemorrhage, although this is more prevalent in elderly individuals and patients with underlying diseases, thrombocytopenia, and coagulation dysfunction. This patient did not have these risk factors, so it is reasonable to consider the possibility of vasculitis caused by EGPA. However, further observation is needed to determine whether heparin has a promoting effect.

## CONFLICT OF INTEREST STATEMENT

The authors declare no conflict of interest.
